# Food Insecurity in the United States: Identification, Treatment, and Implications

**DOI:** 10.1093/geroni/igaa057.2791

**Published:** 2020-12-16

**Authors:** Rose Ann DiMaria-Ghalili, Connie Bales, Julie Locher

## Abstract

Food insecurity is an under-recognized geriatric syndrome that has extensive implications in the overall health and well-being of older adults. Understanding the impact of food insecurity in older adults is a first step in identifying at-risk populations and provides a framework for potential interventions in both hospital and community-based settings. This symposium will provide an overview of current prevalence rates of food insecurity using large population-based datasets. We will present a summary indicator that expands measurement to include the functional and social support limitations (e.g., community disability, social isolation, frailty, and being homebound), which disproportionately impact older adults, and in turn their rate and experience of food insecurity and inadequate food access. We will illustrate using an example of at-risk seniors the association between sarcopenia, the age-related loss of muscle mass and function, with rates of food security in the United States. The translational aspect of the symposium will then focus on identification of psychosocial and environmental risk factors including food insecurity in older veterans preparing for surgery within the Veterans Affairs Perioperative Optimization of Senior Health clinic. Gaining insights into the importance of food insecurity will lay the foundation for an intervention for food insecurity in the deep south. Our discussant will provide an overview of the implications of these results from a public health standpoint. By highlighting the importance of food insecurity, such data can potentially become a framework to allow policy makers to expand nutritional programs as a line of defense against hunger in this high-risk population.

